# The structure of the amygdala associates with human sexual permissiveness: Evidence from voxel‐based morphometry

**DOI:** 10.1002/hbm.22638

**Published:** 2014-09-17

**Authors:** Hikaru Takeuchi, Yasuyuki Taki, Rui Nouchi, Hiroshi Hashizume, Atsushi Sekiguchi, Yuka Kotozaki, Seishu Nakagawa, Carlos Makoto Miyauchi, Yuko Sassa, Ryuta Kawashima

**Affiliations:** ^1^ Division of Developmental Cognitive Neuroscience Institute of Development, Aging and Cancer, Tohoku University Sendai Japan; ^2^ Division of Medical Neuroimaging Analysis Department of Community Medical Supports, Tohoku Medical Megabank Organization, Tohoku University Sendai Japan; ^3^ Department of Radiology and Nuclear Medicine Institute of Development, Aging and Cancer Tohoku University, Sendai Japan; ^4^ Human and Social Response Research Division International Research Institute of Disaster Science, Tohoku University Sendai Japan; ^5^ Research Administration Office Kyoto University Kyoto Japan; ^6^ Department of Functional Brain Imaging Institute of Development, Aging and Cancer, Tohoku University Sendai Japan; ^7^ Japan Division of Clinical Research Medical‐Industry Translational Research Center, Fukushima Medical University School of Medicine; ^8^ Department of General Systems Studies Graduate Schools of Arts and Sciences, The University of Tokyo Bunkyo, Tokyo Japan; ^9^ Department of Advanced Brain Science Smart Ageing International Research Center, Institute of Development, Aging and Cancer, Tohoku University Sendai Japan

**Keywords:** sexual permissiveness, voxel‐based morphometry, gray matter, white matter, amygdala, hypothalamus

## Abstract

Sexual behavior is a critical function of human procreation. Despite previous studies that investigated the neural mechanisms of basic human physiological sexual functions, the neural mechanisms that underlie individual differences in human sexual permissiveness remain unknown. We used voxel‐based morphometry and a questionnaire (scale for sexual attitudes) to measure sexual permissiveness to investigate the gray matter and white matter structural correlates of sexual permissiveness. Sexual permissiveness was negatively correlated with regional gray matter density of the structures involving the right amygdala and surrounding areas, and positively correlated with regional white matter density of the white matter area that spread around the left amygdala to the hypothalamus area. There were no gender‐specific relationships in the neural correlates of our findings. These findings suggest that structural variations in regions that play key roles in the basic physiological aspects of human sexuality underlie individual complex sexual attitudes in social life. *Hum Brain Mapp 36:440–448, 2015*. © **2014 The Authors. Human Brain Mapping Published by Wiley Periodicals, Inc.**

## INTRODUCTION

Sexual behavior is a critical function for procreation in humans as well as in other animals. Sexual relationships consist of emotions, attitudes, and behaviors, and these components are associated with one another in a complex manner [Kaplan, 1980]. It is increasingly accepted that sexual behavior has a substantial emotional component [Sprecher and McKinney, 1993]. Conversely, sexual attitudes have been shown to be multidimensional [Hendrick et al., 2006]. Hendrick and Hendrick [1987] identified four different dimensions of sexual attitudes, and the one that has been considered to be the most important was sexual permissiveness [Hendrick et al., 1985]. Sexual permissiveness typically refers to how far people will go sexually [Hendrick et al., 1985]. Humans exhibit much variability in sexual permissiveness [Hendrick et al., 1985]. The scale for sexual attitudes [Wada and Nishida, 1992] was developed to assess sexual permissiveness together with other aspects of sexual attitudes. The reliability and construct validities of this scale have been well documented [for details, see Methods and Wada and Nishida, 1992].

The investigation of the neural basis of sexual permissiveness is important not only neuroscientifically but also (a) socially because sexual permissiveness is associated with sexually risky behaviors [Wada and Nishida, 1992] and (b) evolutionarily because, in addition to affecting an individual's procreating behaviors, sexual attitudes affect the complex judgment involved in selecting a desirable partner in various situations [Oliver and Sedikides, 1992].

Numerous animal studies have largely clarified the roles played by such subcortical structures as the amygdala and the hypothalamus in sexual behavior [Meisel and Sachs, 1994]. Furthermore, the review of lesion studies of sexual disorders suggests that the amygdala is associated with sexual drive and the hypothalamus is associated with autonomic aspects of sexual drive and so forth [Baird et al., 2007].

However, even though previous studies associated basic human physiological sexual functions with studies of human sexual orientations [Savic and Lindström, 2008], the neural mechanisms that underlies the individual differences in human sexual attitudes and determine complex human attitudes toward various aspects of sexuality in social relationships have not been well investigated.

Here, we hypothesized that structural variation related to the amygdala and the hypothalamus is associated with sexual permissiveness among typically developed young adults. This hypothesis is based on the aforementioned clinical studies that showed that these brain structures are associated with sexual drive and may modulate sexual permissiveness among typically developed young adults.

To test these hypotheses, and to identify the gray matter structural correlates of sexual permissiveness, we investigated how individual differences in sexual permissiveness, as measured by the scale for sexual attitudes, were associated with regional gray matter density (rGMD) and regional white matter density (rWMD) using voxel‐based morphometry (VBM) [Good et al., 2001]. Using an analysis of covariance (ANCOVA), we investigated whether there were any effects of sexual permissiveness on rGMD and rWMD regardless of gender, as well as interaction effects on rGMD and rWMD between the scores of each factor of sexual attitudes and gender.

Empirically, it has been widely established that a wide range of cognitive individual differences in normal adults are reflected in regional gray matter structures [Kanai and Rees, 2011]. As summarized in our previous study [Takeuchi et al., 2012b], potential correlates of GM in VBM may include the number and size of neurons and glial cells, the level of synaptic bulk, and the number of neurites, [May and Gaser, 2006; Takeuchi et al., 2011c]. Structural imaging thus provides unique and distinctive information about the neural origin of individual cognitive characteristics.

## METHODS

### Subjects

Seven hundred and seventy‐seven healthy, right‐handed individuals (433 men and 344 women; 20.7 ± 1.9 years) participated in this study as part of an ongoing project investigating associations among brain imaging, cognitive functions, aging, genetics, and daily habits [Takeuchi et al., 2011a,c, 2014a; 2013a, 2012a, 2010a,b, 2011c; Taki et al., 2010, 2011]. Data derived from the subjects in this study are to be used in other studies irrelevant to the theme of this study. Some of the subjects who participated in this study also became subjects of intervention studies (psychological and imaging data recorded before the intervention were used in this study) [Takeuchi et al., 2013b]. Psychological tests and magnetic resonance imaging (MRI) scans not described in this study were performed together with those described in this study. All subjects were university, college, or postgraduate students or subjects who had graduated from these institutions within 1 year before the experiment and had normal vision. None had a history of neurological or psychiatric illness. Handedness was evaluated using the Edinburgh Handedness Inventory [Oldfield, 1971]. Written informed consent was obtained from each subject in accordance with the Declaration of Helsinki [1991]. This study was approved by the Ethics Committee of Tohoku University.

### Scale for Sexual Attitudes

The scale for sexual attitudes [Wada and Nishida, 1992] was used to assess sexual permissiveness. Before the administration of the questionnaires in this experiment, it was made clear that all subjects had the right to not answer any items in the questionnaires that they did not want to answer. The scale for sexual attitudes is a self‐reported measure of sexual attitudes. The scale was developed and standardized for Japanese subjects. A more detailed discussion of the psychometric properties of this instrument and how it was developed was described by Wada and Nishida [1992] [Wada and Nishida, 1992]. The scale for sexual permissiveness comprises 17 items and uses a five‐point Likert scale with a response format ranging from “I do not think so” to “I think so.” The subject's responses yield the composite scale scores. The ratings for the statements were summed, and negative items were reverse coded.

Sexual permissiveness evaluates the extent of an individual's tendency to be sexually permissive and includes such items as “I can accept a one‐night stand,” “I want to have sexual relationships with various people,” “Prostitution should be admitted in society,” “Having ongoing sexual relationships with a number of people at the same time is acceptable,” and “Sexual conduct is only permissible after marriage (negative item).”

This scale has been normalized based on data from a relatively large sample (*N* = 250) [Wada and Nishida, 1992]. Factor analyses (principal factor method; varimax rotation) extracted the factors of this scale [Wada and Nishida, 1992]. The internal consistencies of the factor of sexual permissiveness are 0.90 (Cronbach's coefficient of *α*) [Wada and Nishida, 1992]. The sexual permissiveness score has been shown to be associated with the extent of an individual's sexual experience [Wada and Nishida, 1992]. These findings show the reliability and external validity of the scale for sexual permissiveness.

### Assessment of Psychometric Measures of General Intelligence

Raven's advanced progressive matrix (RAPM) [Raven, 1998], which is often shown to be the measure most correlated with general intelligence and thus the best measure of general intelligence [Raven, 1998], was used to assess intelligence and adjust for the effect of general intelligence on brain structures [Jung and Haier, 2007]. As seen in the Behavioral data subsection of the Results, sexual permissiveness and RAPM score were not associated. However, because RAPM score has been associated with brain structures [Haier et al., 2004; Jung and Haier, 2007], the inclusion of RAPM score in the model of analyses is intended to improve the accuracy of the model and reduce error variance. For more details of how RAPM was performed, see our previous work [Takeuchi et al., 2010b,c].

### Image Acquisition and Analysis

All MRI data acquisition was conducted with a 3‐T Philips Intera Achieva scanner. Using a MPRAGE sequence, high‐resolution T1‐weighted structural images (240 × 240 matrix, repetition time (TR) = 6.5 ms, echo time (TE) = 3 ms, field of view (FOV) = 24 cm, 162 slices, 1.0 mm slice thickness) were collected. In this project, other types of MRI scans were obtained, but none of the functional magnetic resonance imaging (fMRI) scans involve the tasks that can directly relate to sexual attitudes.

### Preprocessing of T1‐Weighted Structural Data

As described in our previous study [Takeuchi et al., 2012a], both functional imaging studies and structural studies have advantages and disadvantages, but the findings from the two methods should complement each other. Structural imaging studies are especially useful for investigating the anatomical correlates of personal characteristics involving a wide range of behaviors or opinions that occur outside the laboratory, such as sexual permissiveness, because unlike fMRI studies, the results of structural imaging studies are not limited to the specific regions engaged in the task or stimuli during scanning. Furthermore, while fMRI studies have certain apparent advantages, one disadvantage is that simplified everyday tasks have to be performed in an MRI scanner because of the constraints of MRI, and this may lead to different activation patterns in the brain [Okamoto et al., 2004]. Furthermore, in MRI correlation studies (including those of fMRI) that investigated the neural basis of individual differences, we are able to use established cognitive measures with proven reliability and validity to tap individual differences in cognition.

Preprocessing of the structural data was performed using Statistical Parametric Mapping software (SPM8; Wellcome Department of Cognitive Neurology, London, UK) implemented in MATLAB (Mathworks, Natick, MA). It has already been shown that manual volumetry and VBM indeed measure the same things, especially in the subcortical areas [Focke et al., 2014], suggesting the validity of VBM. Using the new segmentation algorithm implemented in SPM8, T1‐weighted structural images of each individual were segmented into six tissues. In this process, the gray matter tissue probability map (TPM) was manipulated from maps implemented in the software so that the signal intensities of voxels with (gray matter tissue probability of the default tissue gray matter TPM + white matter tissue probability of the default TPM) < 0.25 became 0.When this manipulated gray matter TPM is used, the dura matter is less likely to be classified as gray matter (compared with when the default gray matter TPM is used), without other substantial segmentation problems. In this new segmentation process, default parameters were used, except that affine regularization was performed with the International Consortium for Brain Mapping template for East Asian brains. We then proceeded to the diffeomorphic anatomical registration through exponentiated lie (DARTEL) algebra registration process implemented in SPM8. In this process, we used DARTEL import images of the five TPMs from the aforementioned new segmentation process. First, the template for the DARTEL procedures was created using imaging data from 63 subjects who participated in an experiment in our laboratory [Takeuchi et al., 2011b]. Next, using this existing template, the DARTEL procedures were performed for all of the subjects in this study. In these procedures, default parameter settings were used. The resulting images were spatially normalized to the Montreal Neurological Institute (MNI) space to give images with 1.5 × 1.5 × 1.5 mm^3^ voxels. Subsequently, all normalized rGMD and rWMD images were smoothed by convolving them with an isotropic Gaussian kernel of 12 mm full width at half maximum (FWHM) for the reasons described below.

In this study, we used gray matter density instead of gray matter volume as the gray matter index. When using rGMD, VBM can be thought of as a comparison between the relative concentrations of gray or white matter structures in the spatially normalized images. When using regional gray matter volume (rGMV), VBM can be thought of as a comparison of the absolute volume of gray or white matter structures. The results of rGMD and rGMV are usually similar [Good et al., 2001]. To the best of our knowledge, the implications of the (subtle and probably statistically meaningless) difference between these two analyses are not known. Each of these methods has been frequently used in structural studies [Mechelli et al., 2005]. In this study, we used rGMD for two reasons. First, in our recent study, including subjects with the same characteristics as those of this study [Takeuchi et al., 2010a], rGMD across the brain was correlated with emotional intelligence, which represents the individual's emotional and social competence. Because this study addresses complex sexual behaviors in society, rGMD may be associated to a greater extent with sexual attitude. Second, a previous study reported that sexual hormones are associated with gray matter density [Peper et al., 2009]. Conversely, sexual hormones are also associated with some sexual attitudes [Halpern et al., 1994], and rGMD may be associated to a greater extent with sexual attitude in this sense as well.

### Statistical Analyses

We investigated the association between rGMD and rWMD and individual differences in sexual permissiveness. Statistical analyses of morphological data were performed using the VBM5 software, which is an extension of SPM5, for the reasons described below.

In the analyses, we included only voxels that showed rGMD or rWMD values > 0.05 for all subjects. The primary purpose of using thresholds was to cut the periphery of the GM areas and to limit the areas effectively for analyses. We performed this procedure by limiting the areas for analyses to those likely to be GM. The voxels located outside the brain areas are more likely to be affected by signals from outside the brain through smoothing. No other masking procedure was performed.

We investigated the association between rGMD and rWMD and individual differences in sexual permissiveness using whole‐brain ANCOVA. In the whole‐brain analyses, we used voxel‐wise ANCOVA with gender difference as a grouping factor (using the full factorial option of SPM5). In this analytical stage, RAPM score, total intracranial volume (total GM volume + total WM volume + total CSF volume), and the sexual permissiveness score were used as covariates. Age, RAPM score, and the sexual permissiveness score were modeled so that each covariate had a unique relationship with rGMD or rWMD for each gender (using the interactions option in SPM5), which enabled the investigation of the effects of interaction between gender and each covariate. Conversely, total intracranial volume was not modeled in this manner, and a common effect of total brain volume on rGMD or rWMD was assumed for both genders. In these analyses, the centering option was used for centering the interactions. The main effects of the sexual permissiveness score (contrasts of [the effects of the sexual permissiveness score for males, the effects of the sexual permissiveness score for females] were (0.5 0.5) or (−0.5 −0.5)) and the interaction between gender and the average sexual permissiveness score (contrasts of [the effect of the sexual permissiveness score for males, the effect of the average sexual permissiveness score for females] were (−0.5 0.5) or (0.5 −0.5)) were assessed using *t*‐contrasts.

The statistical significance level was set at *P <* 0.05, corrected at the nonisotropic adjusted cluster level [Hayasaka et al., 2004] with an underlying voxel level of *P <* 0.0025. In this nonisotropic cluster‐size test of random field theory, a relatively higher cluster‐determining threshold combined with high smoothing values of more than six voxels leads to appropriate conservativeness in real data. With high smoothing values, an uncorrected threshold of *P* < 0.01 seems to lead to anticonservativeness, whereas that of *P* < 0.001 seems to lead to slight conservativeness [Silver et al., 2010]. We used the VBM5/SPM5 version of this test. This is because a previous validation study of this test using a real dataset [Silver et al., 2010] showed that the conditions of this nonisotropic adjusted cluster size test are very limited and depend on the smoothness of the data, as described above. However, there are substantial differences in the way that SPM8 and SPM5 estimate actual FWHM in the areas analyzed, and this directly affects the cluster test threshold. Therefore, regardless of whether SPM5 or SPM8 is appropriate, our view is that the conditions for this nonisotropic adjusted cluster size test shown by the previous study [Silver et al., 2010] are no longer guaranteed in SPM8, because they are different analyses and produce substantially different results.

### Supplementary Analyses to Investigate the Associations Between Sexual Permissiveness and rGMV and rWMV

In this study, we focused on regional density measure (unmodulated [Ashburner and Friston, 2000] VBM measures), as was the case with our previous studies investigating the associations between socioemotional cognitive differences and regional gray matter structures [Takeuchi et al., 2010a, 2014c]. As described in the Results, we found opposite patterns of correlation between the results obtained for rGMD and rWMD in the bilateral amygdala [rGMD showed a significant (or a tendency for) negative correlation with sexual permissiveness, whereas rWMD showed a significant (or a tendency for) positive correlation with sexual permissiveness]. “‘Non‐modulated' VBM identifies differences in the relative concentration or density of gray or white matter (i.e., the proportion of gray or white matter relative to other tissue types within a region); modulated” VBM identifies differences in volume (i.e., the absolute amount of gray or white matter in different regions)” [Mechelli et al., 2005]. Thus, we reasoned that it could be these results are two aspects of the same phenomenon. Thus, we investigated whether rGMV, rWMV, and both parameters showed a similar pattern in these areas.

In these analyses, all the methods are the same as those in the aforementioned analyses of rGMD and rWMD, except that the dependent variables are rGMV and rWMV images (modulated images to preserve the absolute amount of tissue [Ashburner and Friston, 2000]). The details of the preprocessing methods of these images are mostly described above (and overlap with those of rGMD and rWMD images), and the rest was described in our previous study [Takeuchi et al., 2014b]. We investigated whether the results of the main effects of sexual permissiveness show the same patterns as those of analyses of rGMD and rWMD around areas of the bilateral amygdala.

## RESULTS

### Behavioral Data

Table 1 shows the average, SD, and range of age; the score of RAPM; and scores of sexual permissiveness.

**Table 1:** Mean (SD, range) age, the score of Raven's advanced progressive matrix, and scores of sexual permissiveness

Measure	Males	Females
Age (years)	20.79 (1.97, 18–27)	20.57 (1.67, 18–27)
Raven's advanced progressive matrix	28.92 (3.74, 15–36)	28.28 (3.68, 15–36)
Sexual permissiveness	48.24 (10.70, 17–83)	37.87 (10.01, 17–68)

After correcting for the effect of age and gender, the scores of sexual permissiveness were not significantly correlated with the score of RAPM (*P* > 0.1). Males showed a higher score of sexual permissiveness (*P* < 0.001, *t* = 13.92, two‐tailed *t*‐test). This pattern of gender differences in sexual permissiveness was consistent with that reported in a previous study [Wada and Nishida, 1991].

### Effects of the Sexual Permissiveness Score on rGMD

**Figure 1:**
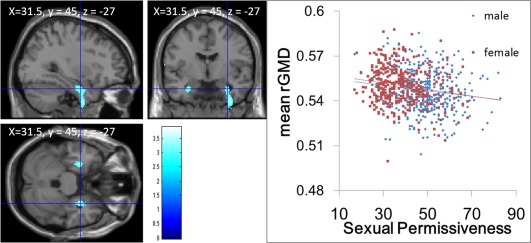
Regions showing a negative correlation (threshold of *P* < 0.0025, uncorrected) between rGMD and the sexual permissiveness score. Regions of correlation are overlaid on a single‐subject T1 image of SPM5. A region of significant correlation is observed in the anatomical cluster that mainly spread around the right amygdalo‐hippocampal complex, together with a tendency for correlation of the right homologue area. Please note that because the results of analyses with a higher smoothing value were overlaid on the unsmoothed single‐subject T1 image for anatomical clarity, the results went slightly beyond the gray matter area of the unsmoothed single‐subject image; however, this does not imply that the results are inadequate. The right panel shows the scatter plots of the associations between mean rGMD of the significant cluster form males and females.

ANCOVA revealed an overall negative main effect (regardless of gender) of the sexual permissiveness score on rGMD in an anatomical cluster that spread mainly in the right amygdalo‐hippocampal complex (Fig. 1, MNI coordinates *x*, *y*, *z* = 36, −1.5, −46.5; *t*‐value of the peak voxel = 3.90; *P* = 0.041 at the nonisotropic adjusted cluster level). A similar pattern was observed in the left homologue area (Fig. 1, MNI coordinates *x, y, z* = −28.5, −4.5, −25.5; *t*‐value of the peak voxel = 3.44; *P* = 0.959 at the nonisotropic adjusted cluster level).

The significance and insignificance of the results of rGMD did not change, when the score of RAPM was removed from the model of the ANCOVA.

No overall significant positive main effect of the sexual permissiveness score was observed on rGMV. No significant effects of the interaction between the sexual permissiveness score and gender were observed on rGMD. As seen in Figure 1, the strength of the association between rGMD in the significant cluster and sexual permissiveness were comparable, too.

### Effects of the Sexual Permissiveness Score on rWMD

**Figure 2:**
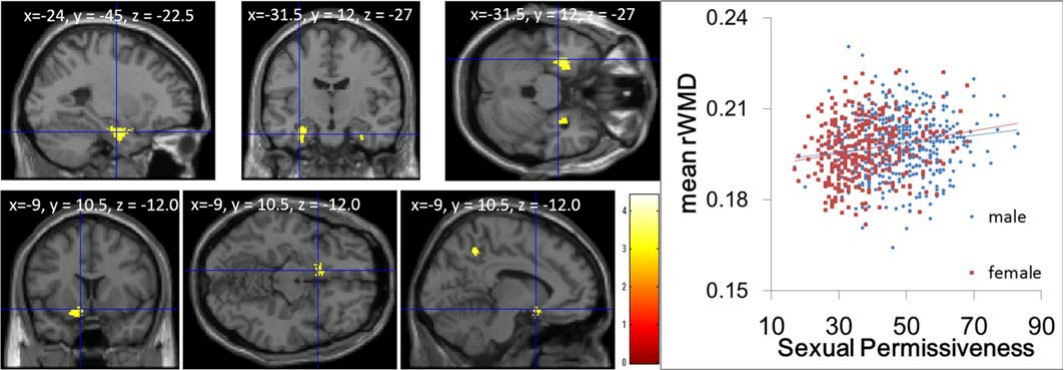
Regions of positive correlation (threshold of *P* < 0.0025, uncorrected) between rWMD and the sexual permissiveness score. Regions of correlation are overlaid on a single‐subject T1 image of SPM5. A region of significant correlation is observed in the anatomical cluster that mainly spread from around the left amygdalo‐hippocampal complex to the area located close to the hypothalamus, together with a tendency for correlation of the white matter area located around the right amygdalo‐hippocampal complex. Please note that because the results of analyses with a higher smoothing value were overlaid on the unsmoothed single‐subject T1 image for anatomical clarity, the results went slightly beyond the white matter area of the unsmoothed single‐subject image; however, this does not mean that the results are inadequate. The right panel shows the scatter plots of the associations between mean rWMD of the significant cluster form males and females.

ANCOVA revealed an overall positive main effect (regardless of gender) of the sexual permissiveness score on rWMD in an anatomical cluster that spread around the amygdala to the hypothalamus area (Fig. 2, MNI coordinates *x, y, z* = −24, −4.5, −22.5; *t*‐value of the peak voxel = 3.82; *P* = 0.046 at the nonisotropic adjusted cluster level). A similar pattern was observed in the area around the right amygdalo‐hippocampal complex (Fig. 2, MNI coordinates *x, y, z* = 34.5, −3, −46.5; *t*‐value of the peak voxel = 4.09; *P* = 0.234 at the nonisotropic adjusted cluster level).

When the score of RAPM was removed from the model of the ANCOVA of rWMD, the statistical significance of the anatomical cluster that spread from the left amygdala to the hypothalamus area weakened (*t*‐value of the peak voxel = 3.54, P = 0.885, corrected). The subtle change in the *t*‐value means the strength of the association itself changed little, and the implication of this change or whether there is meaning, is not clear.

No overall significant negative main effect of the sexual permissiveness score was observed on rWMD. No significant effects of the interaction between the sexual permissiveness score and gender were observed on rWMD. As seen in Figure 2, the strength of the association between rWMD in the significant cluster and sexual permissiveness were comparable, too.

### Supplementary Analyses to Investigate the Associations Between Sexual Permissiveness and rGMV and rWMV

ANCOVA revealed an overall positive main effect (regardless of gender) of the sexual permissiveness score on rWMV in an anatomical cluster that spread around the right amygdala (MNI coordinates *x, y, z* = 36, −4.5, −43.5; *t*‐value of the peak voxel = 3.87; *P* = 0.048 at the nonisotropic adjusted cluster level). A similar pattern was observed in the area around the left amygdalo‐hippocampal complex (MNI coordinates *x, y, z* = −31.5, −9, −28.5; *t*‐value of the peak voxel = 3.36; *P* = 0.532 at the nonisotropic adjusted cluster level). Furthermore, ANCOVA for the negative main effect (regardless of gender) of the sexual permissiveness score on rGMV did not exhibit a pattern that was similar to that of rGMD (not a single voxel showed a negative correlation at the threshold of *P* < 0.01, uncorrected).

As described, “‘non‐modulated' VBM identifies differences in the relative concentration or density of gray or white matter (i.e., the proportion of gray or white matter relative to other tissue types within a region)” [Mechelli et al., 2005]; thus, the results of rGMD and rWMD in and around the bilateral amygdala may be primarily driven by a tendency for increased white matter tissue in the areas located around the amygdala.

## DISCUSSION

To the best of our knowledge, this is the first study that investigated the association between sexual permissiveness and brain structures. As a whole, and consistent with our hypothesis, we revealed that sexual permissiveness was negatively correlated with rGMD of the structures involving the right amygdala and surrounding areas, and positively correlated with rWMD of the white matter area that spread around the left amygdalo‐hippocampal complex to the hypothalamus area. Similar patterns of correlations were observed in the contralateral homologue areas. There were no gender‐specific relationships. These findings suggest that these subcortical structures not only function in the basic physiology of human sexuality but also underlie complex human sexual attitudes in social life. Finally, the patterns of supplemental analyses in the results on rGMV and rWMV suggested the results of rGMD and rWMD observed in regions around the amygdala appeared to be mainly driven by the individual differences of rWMV.

Sexual permissiveness was associated with the subcortical anatomical structures that involve the amygdala and the hypothalamus, both of which are involved in their own way in the neural mechanisms that mediate sexual behavior as described below. The amygdala is activated during sexual arousal [Hamann et al., 2004] and its stimulation can elicit human sexual responses [Gloor, 1986]. Furthermore, this region has been suggested to play an important role in regulating human sexual behavior, specifically sex drive, by regulating the level of emotional significance that individuals attribute to social/sexual cues [Baird et al., 2007]. The hypothalamus is involved in many aspects of physiological mammalian sexuality, such as copulatory behaviors, erection, and ejaculation [Caggiula, 1970; Perachio et al., 1979]. The hypothalamus and the amygdala are strongly interconnected, and these connections are important for their functions [Greenstein and Greenstein, 2000]. Direct white matter pathways from the amygdala to the hypothalamus exist [Lemaire et al., 2011], and the present findings of rWMD appear to correspond to this pathway. Increased development of the brain structures that connect the areas involved in sexuality may lead to their improved functioning regarding their own roles, and may be a driving force behind sexual behaviors. This drive may in turn lead to a greater tendency to welcome sexual behaviors in certain situations (sexual permissiveness).

We can also speculate that the relationship between brain structures and sexual permissiveness may be partly mediated by sexual hormones. It is known that sexual hormones, such as testosterone, are associated with sexual permissiveness and sexual activities [Halpern et al., 1994]. The secretion of testosterone is modulated indirectly by the gonadotropin‐releasing hormone, which is secreted by the hypothalamus [Greenstein and Greenstein, 2000]; these two hormones are part of the same regulatory circuit of hormones [Greenstein and Greenstein, 2000]. However, VBM analyses showed that testosterone level was associated with brain structures in the amygdala [Bramen et al., 2011] and hypothalamus [Neufang et al., 2009] in children, and in these studies, positive associations were observed between rGMV and the testosterone level in these areas. However, the associations between testosterone level and white matter structures or such associations in young adults who showed maturation remain unclear. Perhaps in young adults the developed white matter structures in the hypothalamus and amygdala are affected by the increased number of neurons that secret the gonadotropin‐releasing hormone, or underlain by them, which in turn may lead to a higher baseline testosterone level and higher sexual permissiveness.

This was the first study that investigated the association between brain structures and sexual permissiveness in nonclinical samples. Previous animal and human studies have shown that lesions in brain structures sometimes cause various and serious problems in the sexual behaviors of humans [Baird et al., 2007]. The results of our study suggest that, even in nonclinical samples, structural variations in the areas that involve the amygdala and hypothalamus underlie individual complex sexual attitudes in social life. As sexual attitudes are associated with sexually risky behaviors [Wada and Nishida, 1992], when considering problematic sexual behaviors from a social perspective, one should take into account the fact that subcortical neural systems may underlie the tendency for sexually risky behaviors. From an evolutionary perspective, as sexual attitudes (particularly sexual permissiveness) affect procreating behaviors, as well as an individual's desirability as a partner [Buss, 2000], humans may form individual strategies for procreation [Oliver and Sedikides, 1992]. Our findings indicate that the modulation of subcortical and cortical structures affects the strategies used for procreation.

One of the limitations of this study, similar to our previous VBM studies [Takeuchi et al., 2010a,b], was that we used young healthy subjects with high educational backgrounds. Sexual attitudes may be affected by the level of education [Miller and Sneesby, 1988]. A limited sampling of the full range of intellectual abilities is a common hazard when sampling from college cohorts [Jung et al., 2010]. Whether our findings would also hold across the full range of population samples and normal distribution must be determined using larger and more representative samples. Furthermore, this was not a functional imaging study. Although structural studies have several strong advantages [Takeuchi et al., 2012a], there may be a great disparity between the brain structure and individual tendencies to behave in complex social situations compared with functional imaging studies, in which the actions performed during the cognitive tasks and signals due to brain activation are temporally closely related [Ogawa, 2013]. Thus, we admit that a significant level of speculation and weak reverse inferences were used to discuss some of the findings, as is often the case in this type of structural study involving complex behaviors in real life [Takeuchi et al., 2010a, 2013c], the trade‐off we chose. Future fMRI studies can investigate the manner in which the structures identified here are involved in certain specific cognitions related to complex sexual behaviors.
